# The promise and uncertainty of Medicare's ACCESS model

**DOI:** 10.1093/haschl/qxag018

**Published:** 2026-01-22

**Authors:** Aditya Narayan, Bob Kocher

**Affiliations:** Stanford University School of Medicine, Stanford, CA 94305, United States; Stanford Health Policy, Stanford University School of Medicine, Stanford, CA 94305, United States; Schaeffer Institute for Public Policy & Government Service, University of Southern California, Los Angeles, CA 90089, United States

**Keywords:** Medicare, ACCESS payment model, digital health, healthcare AI, value-based payments

## Abstract

CMS's new ACCESS payment model is a novel approach in how Medicare pays for chronic-disease management: recurring, condition-specific payments tied to clinical improvement, rather than billing for discrete encounters or tightly defined remote-monitoring activities. The promise is straightforward, more reimbursement for technology-enabled longitudinal care and more choice and competition for patient care. But ACCESS leaves many questions unanswered about payment levels, how quality is measured, risk adjusted, and how these patients' facing technology-enabled services coordinate care with the traditional delivery systems. Whether ACCESS strengthens primary care will depend on the details.

## Commentary

More than a decade into the value-based care movement, Medicare's payment-reform efforts remain at an inflection point. The Center for Medicare and Medicaid Innovation (CMMI) was created with the goal of improving care and reducing spending, yet federal evaluations indicate that many of its first 49 models did not achieve sustained savings or measurable improvements in quality, which may not be surprising for a research and development function. A recent Congressional Budget Office analysis concluded that CMMI increased federal spending by more than five billion dollars over its first decade rather than producing the projected savings.^1^ Against this backdrop, the Centers for Medicare and Medicaid Services (CMS) introduced the Advancing Chronic Care with Effective, Scalable Solutions (ACCESS) Model on December 4, 2025. The model's ability to strengthen chronic-disease management and improve the economics of primary care will shape its relevance in the next phase of Medicare payment reform.

The impetus for ACCESS reflects two converging pressures. First, Medicare beneficiaries increasingly require longitudinal management for conditions such as hypertension, diabetes, chronic pain, and behavioral-health conditions, yet fee-for-service mechanisms have been poorly suited to continuous, technology-enabled care. Second, existing codes for remote monitoring and digital engagement have also been narrowly defined and administratively complex—limiting their utility for sustained chronic-disease management. ACCESS aims to address both challenges by creating a reimbursement pathway for such services while tying payment to measurable clinical improvements.^2^

Under ACCESS, participating organizations receive recurring, condition-specific payments to deliver chronic-disease management delivered by digital tools, remote biometric monitoring, app-based behavioral interventions, and virtual care teams. Payments depend on risk-adjusted improvements in validated clinical measures such as blood-pressure control, A1c reduction, pain reduction, or depression response. By focusing on sustained improvement rather than discrete encounters, the model attempts to align payment with the realities of chronic illness in an aging population and to provide the predictable funding.^2^

The model represents a shift in Medicare's economic logic. Although strong evidence links primary care to better outcomes and lower total spending, only 4% to 7% of US health expenditures are allocated to primary care, well below the level observed in other high-income countries.^3^ Within Medicare, spending on longitudinal care is similarly constrained, particularly under definitions that exclude nonphysician clinicians.^4^ Primary care practices, managing rising labor costs and fixed overhead, often struggle to finance the staff, technology, and workflows required for proactive management of chronic conditions. ACCESS seeks to address these pressures by offering recurring upfront payments that are not tied to visit volume.

Payment levels will influence which organizations participate and which care models are feasible. Under ACCESS, organizations receive outcome-aligned payments whose total amount depends on the proportion of patients achieving guideline-informed outcomes. CMS has also outlined a co-management payment of approximately 30 dollars for each documented primary care review, with a one-time 10-dollar onboarding payment and a cap of roughly 100 dollars per beneficiary per year.^2^ These figures stand in contrast to existing remote patient monitoring (RPM) and chronic care management (CCM) codes. National average RPM payments include about 48 dollars per month for treatment management and 43 to 47 dollars per month for device supply and transmission.^5^ CCM pays roughly 60 dollars per month for 20 minutes of care management, with an additional 45 dollars for each extra 20-minute increment.^6^ Relative to these established codes, ACCESS payments are modest, particularly for care models with high labor intensity, reinforcing incentives toward lower-touch, more automated approaches.

Accordingly, a central question is whether the level and structure of ACCESS payments are sufficient to support care models that rely heavily on clinician or care-team time. Models that require frequent synchronous interactions, intensive care coordination, or longitudinal relationship-based support may be difficult to sustain if reimbursement does not adequately cover labor costs. By contrast, approaches that emphasize automation, standardized protocols, and asynchronous engagement may be more economically viable under ACCESS, shaping which types of chronic-care services ultimately scale.

For health systems and clinicians, ACCESS may offer both opportunities and constraints. Large integrated systems already equipped with digital infrastructure, remote-monitoring hubs, and behavioral-health support may be well positioned to implement the model. These organizations can more readily absorb the upfront investment required to redesign workflows and integrate new digital tools. Independent primary care practices, federally qualified health centers, and rural providers, however, may find participation challenging despite the potential benefits of additional chronic-care funding.

For rural and safety-net providers, the challenge is not only whether ACCESS payments are sufficient to support ongoing care delivery, but whether organizations can absorb the upfront fixed costs required to participate at all. Although the model includes a payment adjustment for rural beneficiaries, this adjustment primarily helps offset recurring operating expenses rather than the initial investments needed to build baseline infrastructure. Smaller practices and safety-net institutions may struggle to finance the data analytics, staffing, and technology integration required for participation, even if ongoing payments could eventually support service delivery. As a result, ACCESS may be most accessible to organizations that already possess the necessary infrastructure, raising questions about whether the model can achieve broad participation without complementary support for upfront capacity-building.

The model also has important implications for technology developers. Digital health companies have historically faced obstacles in Medicare markets because time-based remote monitoring codes have been restrictive. ACCESS offers a more predictable mechanism for integrating digital tools into chronic care by tying payment to patient-level outcomes rather than time. Vendors capable of delivering automated or low-touch services at scale may find the economics favorable, while firms that rely on synchronous clinician time or intensive human coaching may struggle to operate under ACCESS alone. This dynamic could shape not only which companies participate but also the extent to which their interventions address the needs of older adults with multimorbidity, limited digital literacy, or high social risk.

For patients, ACCESS could expand access to continuous chronic-disease management, particularly for those with mobility limitations or transportation barriers. Technology-enabled behavioral-health interventions may also help address depression and anxiety, which are common and undertreated among older adults. Yet these benefits will depend on how organizations enroll and support patients. Entities may be more likely to enroll individuals with higher digital readiness or greater likelihood of achieving performance thresholds. Without safeguards, ACCESS could unintentionally widen disparities by making technology-supported care more available to beneficiaries who already have fewer barriers to engagement.

Looking forward, given the early stage of ACCESS, its success will ultimately be determined by a set of questions that have repeatedly emerged in post-mortem evaluations of prior CMMI demonstrations. One critical question is participation and persistence: will ACCESS attract and retain a broad mix of organizations, including small practices and safety-net providers, or will participation concentrate among large, well-capitalized entities with existing digital infrastructure, limiting generalizability and impact? A second question is whether the model produces durable changes in care delivery. Prior CMMI models have often shown modest or transient effects, in part because organizations adapted documentation, coding, or patient selection rather than redesigning longitudinal care. For ACCESS, distinguishing true improvement in chronic-disease management from administrative relabeling will be essential. A third question concerns measurement, risk adjustment, and gaming. Because ACCESS ties payment to outcome improvement, its credibility will depend on whether performance metrics adequately account for medical and social complexity and whether they can detect upcoding, selective enrollment, or shifts in care outside the model's scope. Finally, ACCESS should be judged on whether it improves outcomes that matter to patients without widening disparities. Earlier models struggled to balance cost containment, equity, and broad adoption; for ACCESS, the central test will be whether its payment structure supports meaningful investment in high-need beneficiaries rather than favoring lower-intensity interventions that are easier to scale but less responsive to complex care needs. Answering these questions will require transparent reporting and multiyear evaluation, including attention to implementation costs as well as spending and quality effects.

Moreover, whether ACCESS advances equity will depend on several design choices. Effective risk adjustment will be essential to ensure that outcome expectations do not penalize clinicians caring for patients with high medical or social complexity. Payment levels must also be adequate to support the infrastructure needed for digital engagement and effective care. Over the long term, the sustainability of ACCESS will depend not only on whether payments cover the marginal costs of care delivery but also on whether providers with limited capital can overcome the initial fixed costs required to enter the model. CMS will need to monitor for selective enrollment, differential engagement, and disparities in improvement rates across demographic groups. Smaller practices and safety-net organizations may require targeted support to participate fully. Finally, robust and transparent evaluation will be important for assessing not only average effects but also the distribution of benefits across populations and care settings.

ACCESS reflects a broader recognition that fee-for-service payment is poorly aligned with efficient achievement of better chronic-disease outcomes. If implemented thoughtfully, the model could modernize chronic care, expand access to digital tools, and strengthen the financial foundation of primary care. The degree to which CMS aligns the model's design with equity, feasibility, and sustained investment will shape whether ACCESS becomes a meaningful advance in Medicare payment policy or another demonstration with constrained reach.

## Supplementary Material

qxag018_Supplementary_Data
